# Rapid Prototyping of Slot Die Devices for Roll to Roll Production of EL Fibers

**DOI:** 10.3390/ma10060594

**Published:** 2017-05-29

**Authors:** Alyssa Bellingham, Nicholas Bromhead, Adam Fontecchio

**Affiliations:** Electrical and Computer Engineering Department, Drexel University, Philadelphia, PA 19130, USA; nicholasbromhead@gmail.com (N.B.); fontecchio@coe.drexel.edu (A.F.)

**Keywords:** electroluminescent, 3D-printing, rapid prototyping, light-emitting fibers, roll-to-roll process

## Abstract

There is a growing interest in fibers supporting optoelectrical properties for textile and wearable display applications. Solution-processed electroluminescent (EL) material systems can be continuously deposited onto fiber or yarn substrates in a roll-to-roll process, making it easy to scale manufacturing. It is important to have precise control over layer deposition to achieve uniform and reliable light emission from these EL fibers. Slot-die coating offers this control and increases the rate of EL fiber production. Here, we report a highly adaptable, cost-effective 3D printing model for developing slot dies used in automatic coating systems. The resulting slot-die coating system enables rapid, reliable production of alternating current powder-based EL (ACPEL) fibers and can be adapted for many material systems. The benefits of this system over dip-coating for roll-to-roll production of EL fibers are demonstrated in this work.

## 1. Introduction

The growing popularity of wearable and fabric-integrated technologies has created a need for fibers with unique electronic and electro-optical properties. Research into this area has resulted in fabrics with new abilities including sensing, energy storage, and light emission [1,2,3]. Fibers that emit light via electroluminescence are of particular interest for applications in biomedical monitoring, aerospace, and automotive interior lighting because they are electrically controllable and exhibit fast switching times [4,5,6,7]. The first reports of EL fibers relied on traditional vapor deposition processes which required a special robotic system to rotate the fiber and achieve layer uniformity during deposition [8,9]. The development of solution-processed electroluminescent (EL) material systems has enabled continuous deposition onto cylindrical and fiber-shaped surfaces. 

Dip-coating is the most common technique for fabricating EL fibers from solution-processed materials [4,5,10,11]. However, the dip-coating procedure is heavily reliant on the rheology of the coating material, which includes the viscosity, flow parameters, and solvent evaporation rate [12]. This makes the procedure highly dependent on environmental factors as well as the coating speed and angle. The obstructed opening helps control deposition onto the substrate in slot-die coating, making it easier to accommodate different EL device structures (i.e., layer thickness, number of layers), coating materials, and supporting substrates. There are many advantages of slot-die coating for rapid manufacture of EL fibers including increased production speed, control over coating thickness, and repeatability of coatings, but very few reports have mentioned slot-die coating as the method of EL fiber production. EL fibers are an emerging technology and still face many challenges including emission efficiency, power consumption, brightness, degradation due to environmental factors, flexibility, and sensitivity to substrate surface roughness and handling [4,6,9,13]. With so many factors to consider, demonstrating light emission from a fiber-shaped EL device, even on a small scale, takes precedence over developing roll-to-roll processes for mass production of these fibers. 

This work details the development of a slot die component for a slot die coating system. The uniformity and repeatability of coatings are highly dependent on the characteristics of the slot die component. The die is designed around coating material properties (i.e., viscosity, flow rate, solvent evaporation rate), the coating substrate, and the desired thickness of the final product. However, this design process often requires expensive trial and error adjustments to optimize the die [14]. 3D printing offers a cost-effective alternative for rapid prototyping of slot dies. Here, a CAD model of a cylindrical slot die is presented. This device can be printed with inexpensive plastics to optimize the die for rapid prototyping of EL fiber devices before printing the finalized die in a more expensive material for long term use.

## 2. Design of Coating System

**Figure 1:**
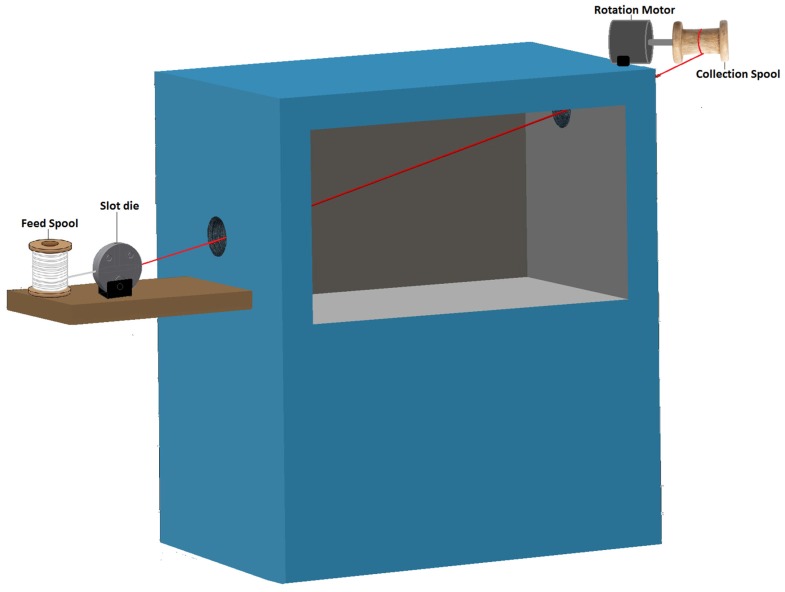
Diagram of automated slot-die coating system illustrating the role and location of the slot die component in the coating process.

The slot die was designed to fit into the automated slot die coating system shown in Figure 1. A modified Blue M isotropic oven with entry and exit points for the yarn to move through was used as the heat source to evaporate the solvent in the coating material. On the entry side of the oven, there is a platform with a spool to hold the uncoated yarn (aka. feed spool), and an adjustable holder that allows the 3D-printed slot die coater to be removed and adjusted to accommodate multiple layer coatings. On the exit side, a collection spool was attached to a motor to continuously pull the yarn through the system at a set speed and collect it on a spool.

### Design of 3D-Printed Slot Die for EL Fiber Production

**Figure 2:**
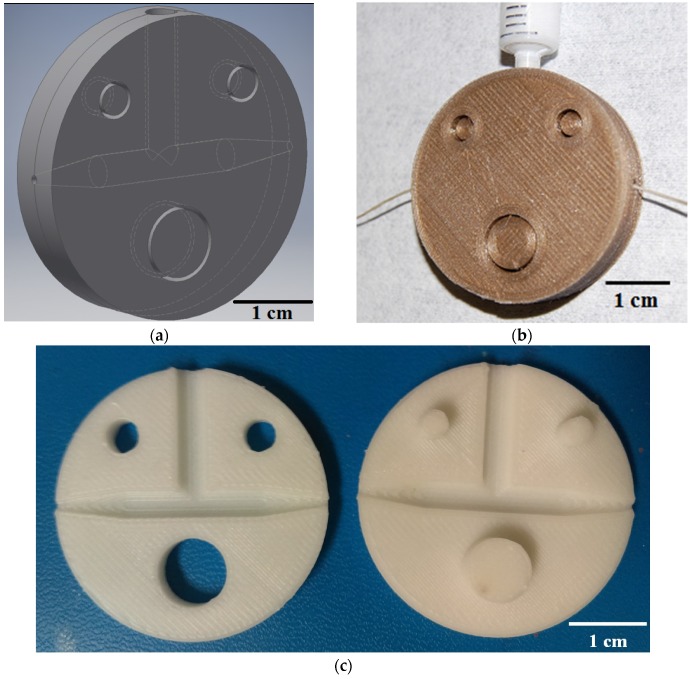
3D-printed slot die coating device. (**a**) Autodesk Inventor^®^ CAD screen capture of the device model; (**b**) Photograph of fiber coating method using device printed with polylactic acid (PLA); (**c**) Photograph of physical device printed with acrylonitrile butadiene styrene (ABS) plastic.

The basic structure of the 3D-printed slot die consists of a fluid chamber that tapers to a small cylindrical opening at both ends, as depicted in the CAD diagram shown in Figure 2a. The die is circular to allow easy adjustment in the automated slot-die system to account for the slight angle the yarn travels at through the system. A fiber substrate is drawn through the fluid chamber containing the coating material, and the obstructed opening controls the thickness of the deposition. The volume of the fluid chamber in this design is small (~403.382 mm^3^), so a standard male luer lock syringe with 4.2 mm opening can be used to continuously flood the chamber during deposition to allow continuous, reliable coating. Figure 2b shows the placement of the fiber coating substrate and fluid coating material entrance locations on the slot die. The cost and time needed to print the 3D slot die devices is dependent upon the amount of printing material used. Thus, the slot die prototypes were kept small to minimize these factors. The slot die device is designed to separate, allowing easy alignment of the fiber inside the fluid chamber. The two-piece disk design is held together by a peg-hole system where one side has three protrusions that match cavities on the opposite disk, as depicted in Figure 2c. All feature dimensions can be adjusted in the 3D model file, making it easy to custom design the slot die for different processes and scale the optimized design.

**Figure 3:**
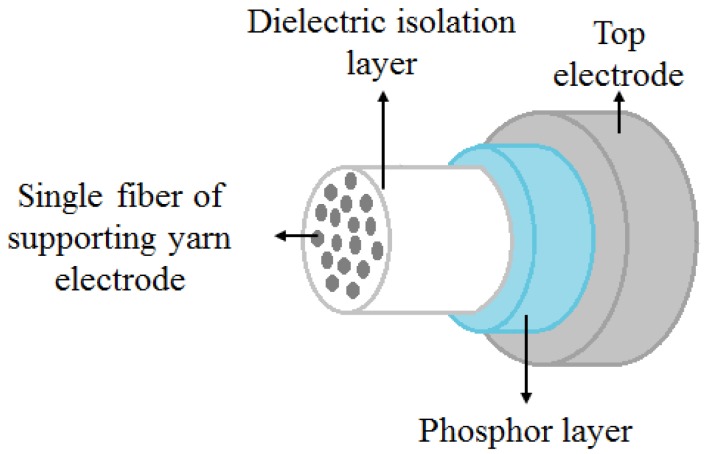
Structure of EL fiber including the supporting conductive fiber (used as on electrode), dielectric isolation layer, phosphor/emitting layer, and the translucent top electrode layer.

An alternating current powder-based electroluminescent (ACPEL) material system was used to produce EL fibers. Solution-processed ACPEL materials have thick layers (on the order of µm) and are flexible, making it simple to deposit onto existing fiber substrates. Currently, it is one of the few EL material systems to produce reliable emission along large area planar and fiber light sources [15,16]. The ACPEL device structure consists of a dielectric isolation layer, an emitting layer, and translucent, conductive top electrode, as shown in Figure 3. During deposition of the isolation layer, the high-dielectric coating material is wicked in between the individual fibers of the conductive yarn acting as the supporting electrode. This creates a monofilament that the other layers can be deposited on.

## 3. Results and Discussion

The thickness of the device layers plays a large role on the optical output of ACPEL devices. The emitting layer consists of phosphor powder suspended in a medium with high dielectric constant to focus the applied field onto the phosphor particles. Luminescence takes place when electrons in the field collide with luminescent impurities in the phosphor via impact excitation and those impurities release photons as they relax down to ground state. Therefore, the light output depends upon the concentration of particles in the device. The doping level of the phosphor material is constant, so a thicker phosphor layer has a greater number of luminescent impurities, which increases the probability of impact excitation. Additionally, a thicker phosphor layer leads to a smaller phosphor capacitance, which results in a higher percentage of the applied voltage being dropped across the phosphor layer.

**Figure 4:**
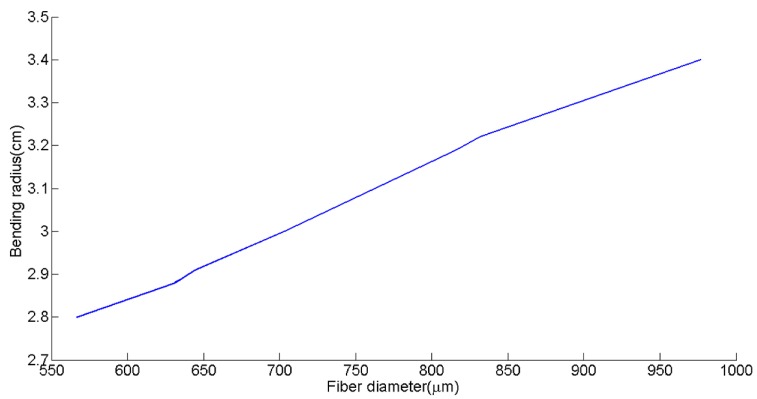
Effect of increasing fiber diameter on fiber bending radius.

Along the same lines, a high insulator layer capacitance with respect to the phosphor capacitance results in a greater percentage of the applied voltage being dropped across the phosphor layer. Thus, a relatively thick phosphor layer and a thin insulation layer was desired to ensure reliable output from the fiber. However, there is a tradeoff between coating thickness and fiber flexibility. The flexibility of the fibers can be quantified by bending radius. Figure 4 demonstrates the nearly linear trend between this fiber diameter increase and the bending radius. The increase in fiber diameter is a result of increasing the thickness of the phosphor layer. Although the bending radius of the fiber improved with thinner layers, reliable light emission from the fibers was the top priority. Therefore, some flexibility was sacrificed to produce robust fibers with brightness visible to the naked eye. The desired layer thicknesses resulted in fibers with a 3 ± 0.05 cm bending radius, which is sufficient to be used in a woven structure or inlaid into a knitted structure.

The EL fiber device layer structure is based on suggested layer thicknesses by the EL material system manufacturer [17]. The desired layer thicknesses are 100 µm for the phosphor layer, 30 µm for the translucent conductive layer, and an average of 30–40 µm for the dielectric isolation layer. The thickness of the coating varies between each individual fiber in the supporting yarn due to the distribution of the dielectric isolation coating material in between the individual fibers during deposition. The system is designed to have an average of 30 µm of dielectric conductive material, separating the emitting layer from the nearest conductive fibers. The individual fibers of the conductive yarn are suspended in the dielectric isolation coating after deposition, thereby creating a monofilament. A similar process is used to design slot dies for the emitting and translucent conductive layers. Table 1 shows the average thickness of each layer produced by the finalized cylindrical slot dies for each layer. 

Most slot dies are made from steel or other non-reactive metals so that they do not react with the 3D printing material. Additionally, these materials conduct heat easily to control the temperature of the fluid bath, and therefore the evaporation rate of the solvent. 3D printing has advanced rapidly over the past few years and machines capable of printing these non-reactive metal materials exist [18]. During the design and testing phase of the slot die system development process, slot die prototypes can be printed with cheap plastics, provided they do not react with the desired coating material. Because the CAD file given to the 3D printer remains the same, it is very simple to translate the design from one material to another. Once the die design is finalized, the design can simply be printed with the chosen metal. This additive manufacturing method of die production is simple and reduces material waste compared to traditional machining procedures [19]. 

### Speed of EL Fiber Production

**Figure 5:**
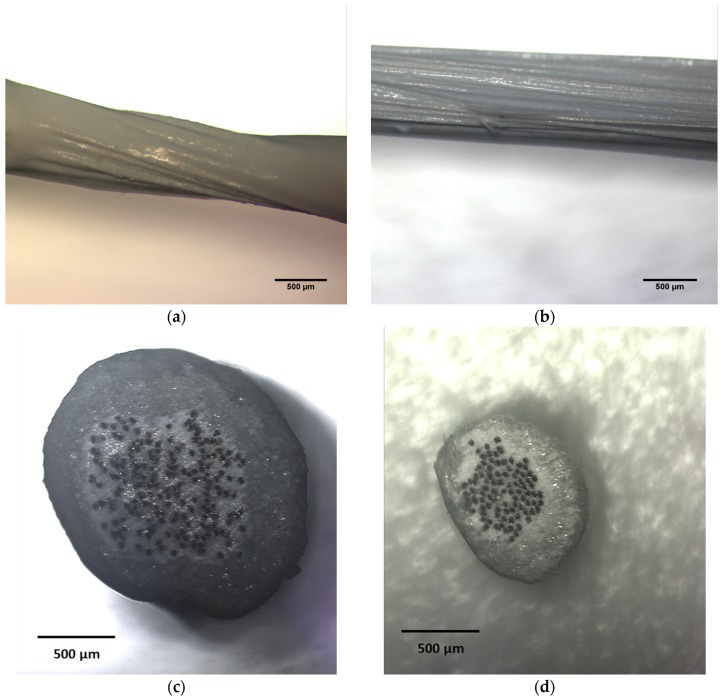
Optical microscope images (5× magnification) of the dielectric isolation layer coating of fibers produced via (**a**) dip-coating and (**b**) slot-die coating. Optical microscope images (5× magnification) of cross sections taken from the center of the (**c**) dip-coated and (**d**) slot-die coated fibers after deposition of the complete EL fiber structure.

The rate and consistency at which EL fibers can be produced becomes increasingly important in scaling manufacturing of the fibers for commercial applications. Slot-die coating offers significant improvement in the rate of fiber production and coating uniformity over dip-coating procedures. Figure 5a,b compare the surface profile of a conductive yarn coated with the dielectric insulation layer via dip-coating and slot-die coating procedures, respectively. It is apparent from these images that the coating thickness and uniformity varied along the dip-coated fibers. The cross sections of the complete EL fiber devices, shown in Figure 5c,d for dip-coated and slot-die coated fibers, respectively, give further insight into the layer thicknesses produced by each method. The emissive layer of dip-coated fibers was, on average, 50 µm thicker than the slot-die coated fiber and exhibited non-uniformity near the ends of the fiber where material tended to pool and create much thicker segments. 

The diameter of the dip-coated fiber core is approximately twice the size of the core of the slot-die coated fibers. This is a side effect of the hand drawing method used to produce the dip-coated fibers. The supporting conductive yarn is the same, however, the fibers are not as closely packed in the dip coated core because they are not fixed at both ends and the fluid isolation layer is randomly dispersed as the fiber is withdrawn from the solution. The fluid chamber in the slot die tapers down to a cylindrical opening on both ends, which forces the fibers into a stricter alignment and significantly decreases the core fiber diameter in these devices. 

Another advantage of the slot-die coating method is the speed at which fibers can be produced. Dip-coating is heavily reliant on gravity and the flow parameters of the fluid coating material [20]. The fluid coating materials have high viscosities in the range of 10–20 Pa·s for the dielectric isolation and phosphor layers and 3.5–10 Pa·s for the translucent conductive layer. Thus, the flow rate for these materials is low and they need to be drawn at a very slow rate from the fluid bath to decrease the thickness of the coating [21]. Equation (1) can be used to estimate this withdrawal speed (*U*_0_) for a desired layer thickness (*h*_0_), where, η is the liquid viscosity, ρ is the liquid density, *g* is the acceleration of gravity, and *c*_1_ is a constant with a value of approximately 0.8 for Newtonian liquids [12,20].

(1) $$U_{0}\;=\;{\left(\frac{h_{0}}{c_{1}}\right)}^{2}\left(\frac{\rho g}{\eta }\right)$$

The speed of coating varies for each EL layer because of the variations in viscosity and desired layer thickness. In slot-die coating, the speed of coating is controlled by the evaporation time of the solvent and total length of fiber that can be cured at a time [14]. However, defects occur in the coating at speeds exceeding the low-flow limit of the coating system, which is reported in Table 2 for each layer in this experimental system [22,23]. In EL fibers, defects in the coating would not only cause non-uniform light emission across the fibers, but could result in the electrode layers contacting each other, creating an electrical short and producing a large amount of heat at those locations. The speed of coating in this experimental system is governed by distance over time and can be estimated by length of fiber cured in a certain amount of time. The cure time is 15 min in air inside a 130° oven for the dielectric isolation and phosphor layers and 5 min at the same temperature for the conductive top layer. In the automated slot-die coating system, 0.368 m of fiber could be exposed to heat at any given time. 

Table 2 compares the withdrawal rates for the dip-coated layers and slot-die coated layers. Slot-die coating is nearly twice as fast as dip-coating for every layer in the ACPEL structure despite using the same fluid coating materials. Slot-die coating is a pre-metered process and the cylindrical die opening controls the layer thickness, whereas dip-coating is entirely dependent on the flow parameters of the fluid coating material in air [20,24]. Thus, slot-die coating acts like an obstructed dip-coating process, allowing precise control over layer thickness and increasing the speed of coating.

## 4. Materials and Methods

### 4.1. EL Fiber Fabrication

The Dupont Luxprint^®^ EL ink material system (Dupont, Wilmington, NC, USA) was used to make robust EL fibers. This system was designed for screen-printing onto flexible planar substrates, so the materials are semi-rigid to maintain their form and are not highly sensitive to handling. The solution-processed layers were deposited in the following order onto the supporting fiber substrate: Dupont Luxprint^®^ 8153 dielectric paste, Dupont Luxprint^®^ 8154L phosphor paste, and Dupont Luxprint^®^ 7164 transparent conductive paste. The dielectric layer fills gaps between the individual silver coated fibers within the underlying conductive yarn, thereby creating a monofilament that subsequent layers are deposited onto. The dielectric and phosphor layers are cured at 130 °C for 15 min, while the translucent conductive paste only takes 5 min to cure at that same temperature. 

Two different methods were used to deposit these layers; dip-coating and slot die coating. In both cases, Aracon^®^ silver-coated kevlar yarn (Micro-coax, Pottstown, PA, USA) was used as the supporting fiber electrode. It was sonicated in a methanol bath for 10 min prior to layer deposition for cleaning. A fiber was immersed in a glass vial full of coating material and hand drawn at an approximate rate of 1 mm/min and angle of 90° for the dip-coating procedure. The fiber was then suspended vertically inside the oven while curing. Slot-die coated fibers were produced by the automated slot-die coating system shown in Figure 1 using slot dies optimized for each layer at the rates given in Table 2. 

### 4.2. Slot Die Fabrication

Autodesk^®^ Inventor (Autodesk, San Rafael, CA, USA) was used to design and edit the CAD model for each slot die. This software can automatically upload designs to the 3D printers available at Drexel University for printing. An Ultimaker 2 3D printer (Ultimaker, Cambridge, MA, USA) with a 0.4 mm nozzle and 100 µm layer height was used to print the devices. Dies in this work were printed with either polylactic acid (PLA) or acrylonitrile butadiene styrene (ABS) because they are inexpensive, readily available, and nonreactive with the EL coating materials.

### 4.3. Characterization Procedures

The bending radius of fibers was determined by affixing the center of a 3-cm segment of fiber to a glass slide and applying pressure to both ends while viewing the center point under 10× magnification using an optical microscope. A ruler underneath the glass slide was used to measure the bend radius as soon as a crack or cracks were visible in the fiber.

The thickness of each layer in the slot-die coated EL fiber was measured at 10 different locations on a circular cross section. Ten different cross sections were taken at points spaced 30 mm apart along the length of fiber produced in the automated slot-die coating system. The average thickness and standard deviation for each layer are based on 100 data points. 

## 5. Conclusions

In this work, slot dies were produced using additive manufacturing to fit into an automated slot-die coating system for roll-to-roll production of electroluminescent fiber. The use of slot die coating to deposit layers of an ACPEL device onto a yarn substrate significantly improved coating uniformity over traditional dip coating, which plays an important role in device performance. The slot-die coating method is often faster than dip-coating because the thickness can be controlled by the slot die opening instead of relying upon gravity and fluid flow parameters. In the case of the ACPEL material set used to produce to light-emitting fibers, slot-die coating is over twice as fast as dip-coating. This system enables rapid manufacturing of EL fiber for fabric-integrated lighting.

## Figures and Tables

**Table 1:** Average layer thickness produced by final cylindrical slot die design.

Layer	Cylindrical Die Diameter (mm)	Average Thickness (µm)
Dielectric isolation layer	1.2	37.24 ± 5.97
Emitting layer	1.36	98.76 ± 6.32
Transparent conductive layer	1.42	32.92 ± 11.89

**Table 2:** Comparison of coating method withdrawal speed for layers in ACPEL structure.

Layer	Desired Layer Thickness (µm)	Dip-Coating Speed (m/h)	Slot-Die Coating Speed (m/h)
Insulation	40	0.625	1.472
Emitting	100	0.951	1.472
Translucent conductive	30	0.312	4.416
